# The ratio of CD8 + lymphocytes to tumor-infiltrating suppressive FOXP3 + effector regulatory T cells is associated with treatment response in invasive breast cancer

**DOI:** 10.1007/s12672-022-00482-5

**Published:** 2022-04-19

**Authors:** Noriko Goda, Shinsuke Sasada, Hideo Shigematsu, Norio Masumoto, Koji Arihiro, Hiroyoshi Nishikawa, Shimon Sakaguchi, Morihito Okada, Takayuki Kadoya

**Affiliations:** 1 Department of Surgical Oncology, Research Institute for Radiation Biology and Medicine, Hiroshima University, Hiroshima, Japan; 2 Department of Breast Surgery, National Hospital Organization Kure Medical Center and Chugoku Cancer Center, Kure, Japan; 3 Department of Anatomical Pathology, Hiroshima University, Hiroshima, Japan; 4 Experimental Immunology, WPI Immunology Frontier Research Center, Osaka University, Osaka, Japan; 5 Division of Cancer Immunology, Exploratory Oncology Research and Clinical Trial Center, National Cancer Center, Chiba, Japan; 6 Department of Immunology, Nagoya University Graduate School of Medicine, Nagoya, Japan

**Keywords:** Breast cancer, Regulatory T cell, Tumor-infiltrating lymphocyte, FOXP3, CD8, Neoadjuvant chemotherapy

## Abstract

**Purpose:**

FOXP3 + and CD8 + are recognized markers of tumor-infiltrating lymphocytes (TILs) for breast cancer. FOXP3 + TILs are composed of effector Tregs (eTregs) and other subpopulations that are classified by their differences in suppressive function. In this prospective study, we evaluated Treg subpopulations and CD8 + TILs in breast cancer.

**Methods:**

84 patients with breast cancer were enrolled. Fresh TILs were extracted andTregs were classified into eTregs (CD4^+^FOXP3^high^CD45RA^−^), other FOXP3^+^ Treg subsets (naïve and non-Tregs), and total CD8^+^CD4^−^ TILs using flow cytometry. The suppression strength of each Treg subpopulation was analyzed. The association between TIL subpopulations, clinicopathological characteristics, and response to chemotherapy was evaluated.

**Results:**

The mean CD8/eTreg ratio value was 7.86 (interquartile range: 4.08–12.80). The proliferation function of eTregs was significantly suppressed compared with that of the other subpopulations (proliferation rates: control: 89.3%, + naiiveTreg: 64.2%, + non-Treg: 78.2% vs eTreg 1.93%; all P < 0.05). The patients with high with a high CD8 + /eTreg ratio achieved excellent pathological complete response (pCR) rate of neoadjuvant chemotherapy (90.2%) and the CD8/eTreg ratio were independent predictive factors for pCR (odds ratio:18.7(confidence interval 1.25–279) P < 0.05). A detailed assessment of the CD8/eTreg ratio for each patient who underwent NAC revealed that high CD8/eTreg ratio showed a significantly higher pCR rate compared to patients with a low CD8/FOXP3 ratio (39.6% vs 13.3, P < 0.05) in triple negative subtype patients with stromal TILs < 50%.

**Conclusions:**

A high CD8/eTreg ratio enhances pCR rate in patients with invasive breast cancer.

## Introduction

Breast cancer is a common malignancy that leads to morbidity and mortality in women worldwide [1–3]. According to the Global Cancer Statistics report in 2021, approximately 2.2 million women were diagnosed with breast cancer with nearly 700,000 deaths [2].

Several studies have shown that tumor-infiltrating lymphocytes (TILs) in breast cancer are strongly associated with treatment response and patient prognosis [4–7]. TILs are typically quantitatively evaluated using hematoxylin–eosin (HE)-stained samples. Subsequently, qualitative assessments are performed as TILs are functionally heterogeneous and consist of immunoprogressive or immunosuppressive components [8]. Regulatory T cells (Tregs), the most representative immunosuppressive TILs, are positive for transcription factor forkhead box P3 (FOXP3) and regulate anticancer immunity [9–11]. Some studies have reported increased Tregs in breast cancer as an adverse prognostic factor [12–15]. In contrast, other studies have suggested that Tregs predict favorable outcomes [16–19]. Thus, the role of Tregs in breast cancer TILs remains controversial [20]. Meanwhile, CD8 + TILs are representative immunoprogressive TILs and have been confirmed as an independent predictive factor for treatment response [21–23] or survival [24]. Furthermore, several studies recommend the evaluation of the CD8 + /FOXP3 + ratio of TILs as sensitive markers of tumor immune responses in breast cancer rather than evaluation of FOXP3 + or CD8 + TILs alone [25–29]. These studies indicate that the balance of TIL components, which have conflicting functions, influence the prognosis of breast cancer.

FOXP3, detected by immunohistochemical staining, is a general Treg marker. However, FOXP3 + cells are functionally heterogeneous and can be classified into three fractions using flow cytometry based on the expression levels of FOXP3 and naïve T cell marker CD45RA: naïve Treg, effector Treg (eTreg), and non-Treg. Only eTreg cells have a suppressive function; the other fractions are nonsuppressive and secrete inflammatory cytokines [10, 30]. In colorectal cancer, it has been reported that the variation in the tumor-infiltrating Treg component is caused by specific chemokines and cytokines and that these variations affect disease prognosis [31]. eTregs were recently revealed using dual immunostaining of FOXP3 and CTLA4 in diffuse large B cell lymphoma [32]. However, the variations in eTreg infiltration have scarcely been evaluated in breast cancer. Thus, the role or significance of Treg subpopulations in breast cancer remains unclear. Herein, we examined the association between eTregs and CD8 + TILs and the clinical outcomes of patients with invasive breast cancer.

## Materials and methods

### Patients and treatments

This prospective study enrolled 84 patients with early breast cancer who underwent complete resection between December 2015 and November 2016 from department of breast surgery, Hiroshima university hospital. Male patients and patients with noninvasive or microinvasive carcinoma, rather than primary breast cancer, were excluded from the study. The protocol of this study was approved by the Ethics Committee of Hiroshima University and was conducted in accordance with the Declaration of Helsinki. Written informed consent was obtained from all patients.

The Neoadjuvant chemotherapy (NAC) regimen consisted of four cycles of docetaxel (75 mg/m^2^, every 3 weeks), followed by four cycles of FEC (500 mg/m^2^ 5-fluorouracil, 100 mg/m^2^ epirubicin, and 500 mg/m^2^ cyclophosphamide; every 3 weeks). Patients with human epidermal growth factor receptor 2 (HER2)-positive breast cancer received trastuzumab (8 mg/m^2^ for the first dose and 6 mg/m^2^ thereafter) every 3 weeks together with docetaxel. Pathological compete response (pCR) was defined as the absence of invasive residuals in the primary lesion and axillary lymph nodes [33].

### Breast cancer tissue collection and extraction of TILs

Fresh samples of invasive breast cancer tissues were collected via core needle biopsy, vacuum-assisted biopsy (Mammotome Elite; Mammotome, Cincinnati, OH, USA), or surgery. Biopsy specimens from patients who had received NAC were collected before treatment. Tumor samples were collected as follows: three to five samples using 16-gauge biopsy needles, > 6 samples using 13-gauge Mammotome needles, or an area of at least 10 mm × 10 mm × 2 mm shaved using a razor during surgery. Fresh TILs were extracted as described previously [31]. Fresh tissues were rapidly cut into small pieces using tissue scissors and homogenized using a GentleMACS dissociator (Miltenyi Biotech, Bergisch Gladbach, Germany), and the TILs were collected from the cell suspensions. For control samples, lymphocytes from normal breast tissue (LNBT) were collected from three patients. Peripheral blood mononuclear cells (PBMCs) from three patients were prepared using Ficoll gradient centrifugation as described previously [31].

### Flow cytometry

Fresh TILs were washed with phosphate-buffered saline containing 2% fetal calf serum and allophycocyanin-conjugated anti-CD4 mAb (BD Biosciences, Franklin Lakes, NJ, USA), V500-conjugated anti-CD8 mAb (BD Biosciences), fluorescein isothiocyanate-conjugated anti-CD45RA mAb (BD Biosciences), and Fixable Viability dye (eBioscience, San Diego, CA, USA). Intracellular staining of FOXP3 was performed using an anti-FOXP3 mAb and FOXP3 Staining Buffer Set (eBioscience) according to the manufacturer’s instructions. The cells were analyzed using LSRFortessa (BD Biosciences) and FlowJo software (Tree Star, Ashland, OR, USA).

### Determination of TIL subpopulations

TILs were categorized according to the methods described in a previous report [8, 28]. TILs were gated into CD4 − CD8 + T cells and CD4 + CD8˗ T cells, and the CD4 + CD8˗ T cell fraction was further gated based on FOXP3 and CD45RA expression as follows: naïve Treg, FOXP3lowCD45RA + ; eTreg, FOXP3highCD45RA˗; and non-Treg, FOXP3lowCD45RA˗. The ratio of each TIL subpopulation to the total CD4 + CD8˗ TIL population was measured. PBMCs and LNBT were used as a control for validation.

### Assessment of suppressive strength in each Treg subpopulation

Treg subpopulations (naïve Treg, eTreg, and non-Treg cells) were isolated from TILs using FACS Aria II (BD Biosciences) using a previously reported protocol [28]. Briefly, 1 × 10^4^ CFSE(carboxyfluorescein diacetate succinimidyl ester)-labeled responder CD25 − CD45RA + CD4 + T cells isolated from the PBMCs were cocultured with 1 × 10^4^ unlabeled Treg subpopulations (naïve Treg, eTreg, and non-Treg cells). Meanwhile, 1 × 10^5^ irradiated autologous accessory cells were stimulated with 0.5 μg/mL plate-bound anti-CD3 (OKT3 mAb) antibody in a 96-well round-bottom plate. Proliferation of CFSE-labeled cells was assessed using flow cytometry after 5 days of culture. The proliferation rate of responder T cells was calculated by dividing the number of proliferating CFSE-diluting responder cells in the presence of each Treg subpopulation at a 1:1 ratio by the number of responder cells prior to culture and multiplying the result by 100.

### Pathological assessment and evaluation of stromal TILs

Histological characteristics, such as histology, nuclear grade, estrogen receptor (ER) and HER2 status, Ki-67 labeling index, and stromal TILs, were assessed by two pathologists. ER and HER2 were assessed according to the American Society of Clinical Oncology/College of American Pathologists Guidelines, in which the molecular subtypes of invasive breast cancer are classified as luminal (ER + HER2), HER2 (ER ± HER2 +), or triple-negative (TN) (ER-HER2-) [34]. The Ki-67 labeling index was scored as high (≥ 20%) or low (< 20%). Stromal TILs were assessed on HE-stained slides of maximum number of tumor lesions using the methodology proposed by the 2014 International TILs Working Group [35]. Lymphocyte-predominant breast cancer (LPBC) was defined as stromal TIL ≥ 50%.

### Statistical analyses

Basic statistics for the TIL subpopulations are expressed as the median and interquartile range (IQR). Analysis of variance was used to compare different factors between groups. The Wilcoxon rank-sum test was performed for multiple pairwise comparisons. Receiver operating characteristic (ROC) curve analysis was used to evaluate the cut off value of each TIL parameter. The sensitivity, specificity, and accuracy (with 95% confidence intervals) were calculated, and the optimal threshold was determined using the Youden index. Statistical significance was set at P < 0.05. All statistical analyses were performed using JMP Pro14 SAS software (SAS Institute Inc., Cary, NC, USA).

## Results

**Fig. 1:**
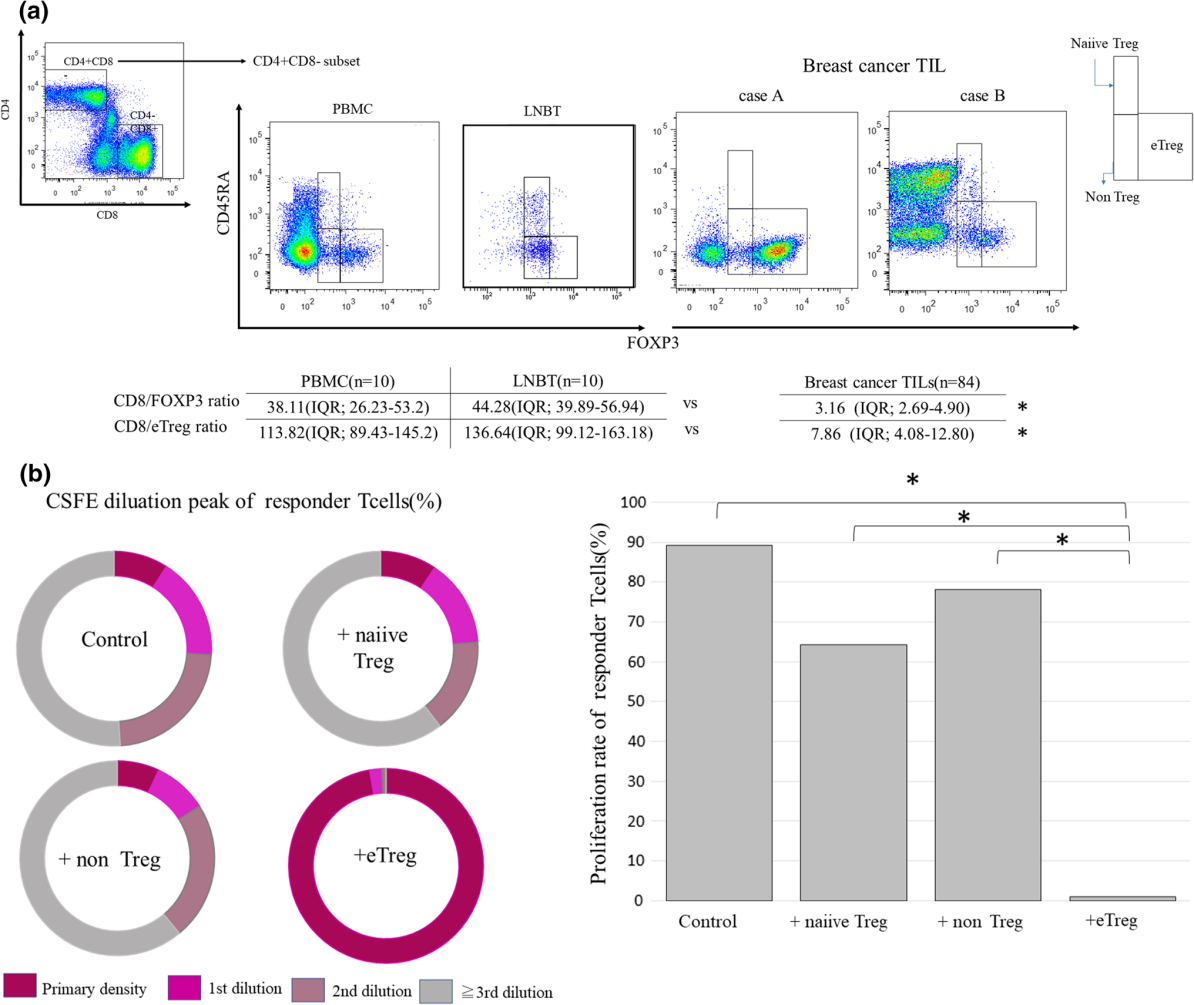

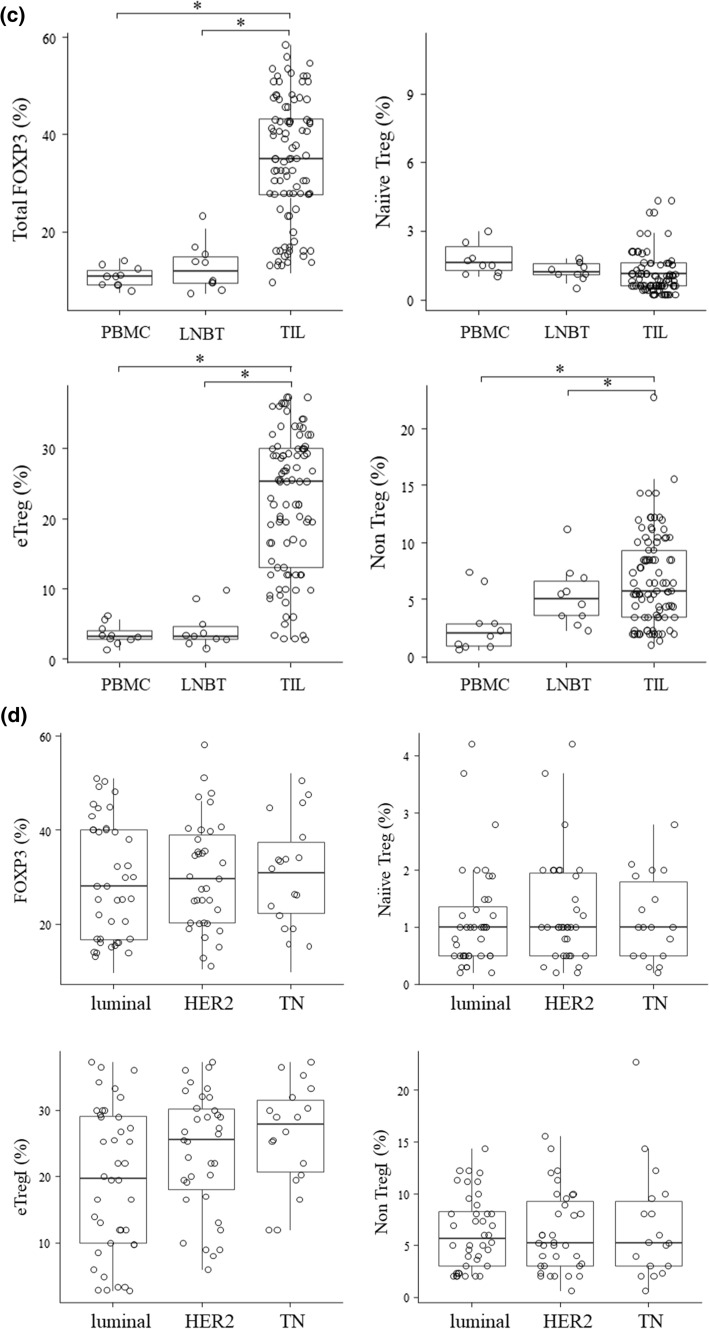
Treg subpopulations as categorized using flow cytometry. **a** CD4^−^CD8^+^ T cells and CD4^+^CD8^−^ T cells were separated using flow cytometry. CD4^+^CD8^−^ cells were further classified into naïve Treg, eTreg, and non-Treg cells using FOXP3 and CD45RA staining. The TIL subpopulations in LPBC, LNBT, and breast cancer TILs are shown in the center. CD8/FOXP3 and CD8/eTreg ratios were calculated using the proportions of these subpopulations. The values of PBMC (n = 10) and LNBT (n = 10) are shown as the median value (IQR). Breast cancer TILs (Cases A and B) are shown as representative samples, and the CD8/FOXP3 and CD8/eTreg ratios are calculated. **b** In vitro suppression assay of breast cancer TILs. CFSE labeled CD4 + CD25- responder T cells from PBMC cultured alone (control) or with naïve Treg, eTreg, or non-Treg cells sorted from freshly extracted breast cancer TILs were cocultured at a 1:1 ratio for 5 days. The proliferation peaks of each group following CSFE dilution were counted (right) and the proliferation rates were calculated (left). These are representative of three independent experiments; each value is the median value. **c** The proportion of total FOXP3 + cells and that of the Treg subpopulation of PBMC(n = 10), LNBT(n = 10), and breast cancer TILs(n = 84). % indicates the ratio of CD4 + T cells. **d** The proportion of total FOXP3 + cells and that of the Treg subpopulation of breast cancer TILs according to molecular subtypes. The data of Luminal (n = 44), HER2(n = 28) and TN(n = 20) subtype cases and were demonstrated by each value (dot) median(bar), and IQR(square)

Table 1 summarizes the clinicopathological characteristics of 84 patients with invasive breast cancer. Among them, 32 patients (32.2%) had nodal metastasis and 25 (29.8%) had lymphocyte predominant breast cancer (LPBC). In total, 50 patients (59.5%) had ER-positive diseases and 28 (33.3%) were HER2-positive. A total of 39 patients received NAC and 20 (51.3%) achieved pCR. Among the fresh TILs, the median number of total TILs was 6.9 × 10^5^ (IQR: 1.2–82 × 10^5^) cells, which was considered sufficient for the analyses. CD4^+^CD8^−^ T cells were categorized as eTregs, naïve Tregs, and non-Tregs via flow cytometric analysis for CD45RA and FOXP3 expression. CD8/FOXP3 and CD8/eTreg values were calculated for these populations. Both CD8/FOXP3 and CD8/eTreg values were significantly decreased in breast cancer TILs compared to those of PBMC and LNBT (CD8/FOXP3 ratios: PBMC 38.11 [IQR: 26.23–53.2], LNBT 44.28 [IQR: 39.89–56.94] vs breast cancer TILs 3.16 [IQR: 2.69–4.90]; CD8/eTreg ratios: PBMC 113.82 [IQR: 89.43–145.2], LNBT 136.64 [IQR: 99.12–163.18) vs breast cancer TILs 7.86 [IQR: 4.08–12.80], all P < 0.05) (Fig. 1a).

**Table 1:** Patient characteristics

	Number (%)
Age (year), median (range)	57 (33–83)
Histological type	
Infiltrating duct carcinoma	75 (88.2)
Lobular carcinoma	6 (7.0)
Other	3 (3.5)
T status	
T1	22 (26.1)
T2	54 (64.2)
T3	4 (4.7)
T4	4 (4.7)
Nodal metastasis	
Negative	52 (61.9)
Positive	32 (38.1)
Nuclear grade	
1	4 (4.8)
2	27 (32.1)
3	53 (63.1)
LVI positive	31 (36.9)
ER positive	50 (59.5)
HER2 positive	28 (33.3)
Ki-67 labeling index	
< 20%	20 (23.8)
≥ 20%	64 (76.2)
Stromal TILs	
Non-LPBC (stromal TIL < 50%)	59 (70.2)
LPBC (stromal TILs ≥ 50%)	25 (29.8)
Neoadjuvant chemotherapy	39 (46.4)
Non-pCR	19 (48.7)
pCR	20 (51.3)

*ER* estrogen receptor, *HER2* human epidermal growth factor receptor 2, *LPBC* lymphocyte predominant breast cancer, *LVI* lymphovascular invasion, *pCR* pathological complete response, *TILs* tumor-infiltrating lymphocytes

The suppression strength of each Treg subpopulation was evaluated. The CSFE labeled-responder T cells cultured alone (the control), naïve Treg, and non-Treg subpopulations showed several proliferation peaks following CSFE dilution. Few proliferation peaks were identified in the CSFE labeled-responder T cells cocultured with + eTregs, indicating their strong immunosuppressive function. The proliferation rate of the responder T cells cultured alone (the control), naïve Tregs, and non-Tregs was significant compared to that of eTregs (proliferation rates: control: 89.3%, + naïveTreg: 64.2%, and + non-Treg: 78.2% vs eTreg 1.93% all P < 0.05) (Fig. 1b).

The proportion of eTregs and non-Tregs among CD4 + TILs was significantly higher in breast cancer TILs compared to those of PBMC or LNBT (mean eTreg of breast cancer TILs: 25.6% [IQR: 312.2–29.1%] vs PBMC: 4.2% [IQR: 3.5–5.2%] and LNBT: 4.0% [IQR: 3.9–5.1%]; mean non-Treg of breast cancer TILs: 5.2% [IQR: 3.2–9.8%] vs PBMC: 2.4% [IQR: 2.1–3.1%] and LNBT: 4.9% [IQR: 3.6–7.1%]) (Fig. 1c). No significant differences among the biomarker subtypes of tumors (ER + , HER2( +), and TN) were found in any breast cancer TIL Treg subpopulation (Fig. 1d). The median percentage of CD8^+^ TILs was 124% (IQR: 87.5–140). Table 2 shows the association of further clinicopathological features with FOXP3^+^, eTregs, and CD8^+^TILs. CD8^+^TILs were significantly associated with HER2 amplification(HER2-:113.0% [IQR:83.8–131.0%]vs HER2 + 130.5% [IQR: 93.3–192.8%], P < 0.05) and LPBC(non LPBC:155% [IQR:92.0–213.5%]vs LPBC:110.0% [IQR: 86.0–128.8%], P < 0.05).

**Table 2:** Relationship between clinicopathological features and TIL subpopulations

	n	FOXP3^+^		Naïve Treg		eTreg		Non-Treg		CD8^+^	
		Median (IQR)	*P*	Median (IQR)	*P*	Median (IQR)	*P*	Median (IQR)	*P*	Median (IQR)	*P*
Age											
< 50 year	28	31.7 (24.9–38.8)	0.913	1.7 (0.8–4.2)	0.970	21.2 (15.6–26.5)	0.605	6.7 (3.9–10.0)	0.239	113.0 (93.3–141.0)	0.849
≥ 50 year	56	30.7 (24.2–40.9)		2.0 (0.9–3.3)		20.1 (16.5–24.9)		7.8 (5.0–13.1)		116.0 (86.0–140.5)	
T status											
T1	22	34.8 (25.5–40.3)	0.401	1.9 (1.0–4.1)	0.935	22.4 (18.0–26.0)	0.344	8.3 (5.0–12.7)	0.558	113.5 (76.5–153.5)	0.665
T2–4	62	30.0 (24.4–40.3)		2.0 (0.9–3.3)		19.9 (16.1–25.5)		7.3 (4.5–11.2)		115.5 (89.5–139.5)	
Nodal metastasis											
Negative	52	30.7 (23.8–38.8)	0.289	2.0 (1.0–3.3)	0.971	20.0 (15.7–24.9)	0.384	7.7 (4.5–11.8)	0.751	117.0 (86.0–150.3)	0.522
Positive	32	32.3 (26.0–42.7)		2.0 (0.8–3.8)		21.4 (16.6–28.6)		7.3 (5.0–11.1)		108.5 (88.0–133.0)	
Nuclear grade											
1–2	31	33.8 (24.5–42.6)	0.347	2.2 (0.8–4.2)	0.307	20.0 (13.0–26.8)	0.792	7.3 (5.3–14.3)	0.191	113.0 (86.0–128.0)	0.319
3	53	29.3 (24.5–38.7)		1.9 (1.0–3.2)		20.3 (16.5–25.4)		7.3 (4.1–10.6)		117.0 (87.0–154.0)	
LVI											
Negative	57	30.7 (24.7–39.4)	0.427	2.0 (1.0–3.8)	0.457	20.0 (14.8–23.0)	0.193	7.1 (4.5–10.0)	0.292	117.0 (87.0–147.5)	0.515
Positive	27	33.8 (23.7–42.0)		1.8 (0.8–3.2)		22.0 (16.5–29.0)		8.8 (5.5–12.2)		104.0 (86.0–132.0)	
ER											
Negative	34	28.7 (25.7–38.2)	0.417	1.5 (0.7–2.8)	0.084	20.3 (16.1–25.8)	0.971	7.3 (4.8–9.9)	0.384	114.0 (90.5–153.5)	0.444
Positive	50	32.6 (23.8–41.9)		2.0 (1.1–4.1)		20.1 (16.2–25.5)		7.5 (4.8–14.2)		115.0 (85.8–138.3)	
HER2											
Negative	56	32.7 (25.1–40.9)	0.553	2.0 (0.9–3.8)	0.296	20.2 (14.2–25.4)	0.439	7.8 (5.3–14.0)	0.028	113.0 (83.8–131.0)	0.031
Positive	28	28.7 (23.2–38.7)		1.9 (0.5–2.7)		20.2 (17.1–26.5)		5.7 (3.5–9.8)		130.5 (93.3–192.8)	
Ki-67 labeling index											
< 20%	20	33.3 (26.0–40.6)	0.834	1.6 (0.5–4.2)	0.436	20.2 (13.0–26.1)	0.896	7.1 (5.1–15.1)	0.501	108.5 (87.5–131.8)	0.386
≥ 20%	64	30.7 (24.2–40.1)		2.0 (1.0–3.3)		20.2 (16.5–25.5)		7.5 (4.0–11.3)		117.0 (86.5–150.3)	
Stromal TILs											
Non-LPBC	59	36.7 (25.5–42.4)	0.136	1.9 (0.9–2.9)	0.769	20.8 (18.8–26.4)	0.178	9.8 (5.1–15.4)	0.193	155.0 (92.0–213.5)	0.002
LPBC	25	30.6 (24.1–38.3)		2.0 (0.9–3.9)		19.9 (13.0–25.5)		7.3 (4.6–10.0)		110.0 (86.0–128.0)	

*ER* estrogen receptor, *HER2* human epidermal growth factor receptor 2, *LPBC* lymphocyte predominant breast cancer, *LVI* lymphovascular invasion, *TILs* tumor-infiltrating lymphocytes

The therapeutic responses of the 39 patients receiving NAC were evaluated. The cut-off values that predict pCR were used to stratify patients into groups with high or low levels of each parameter (eTregs, 9.1% CD4^+^ cells: high n = 20, low n = 19; CD8^+^, 113% of CD4^+^ cells: high n = 18, low n = 21; CD8^+^/eTreg, 13.3: high n = 13, low n = 26; CD8^+^/FOXP3^+^, 4.6: high n = 12, low n = 27). Patients with TN, HER2 subtypes achieved higher pCR rates than those with the luminal subtype (P < 0.05). Patients with LPBC achieved higher pCR rates than those with non-LPBC (P < 0.05). There was no significant difference in the pCR rate between patients with high and low eTregs. Patients displaying a high CD8^+^/eTreg ratio achieved significantly higher pCR than those displaying a low CD8^+^/eTreg ratio (P = 0.001). In contrast, pCR rates did not differ significantly according to CD8^+^/FOXP3^+^ ratio (Table 2). We evaluated specific cilinicopathological factors predicting pCR (Table 3). LPBC and the presence of a high-CD8 tumor were significant predictors of pCR (P = 0.006 and P = 0.020, respectively). After investigating the balance of CD8 + CTLs and Treg subpopulations, the CD8/eTreg ratio was identified as the most promising parameter for predicting pCR, with a positive predictive value of 91.7%. In the multivariate analysis, both LPBC and a high CD8/eTreg ratio were independent predictors for pCR (odds ratio [OR] 10.1, P = 0.043 and OR 18.7, P = 0.034, respectively) (Table 4).

**Table 3:** Pathological response according to tumor subtype and TIL subpopulation

	Non-pCR	pCR	*P*
	19 (48.7)	20 (51.3)	
ER			0.056
Negative	7 (33.3)	14 (66.7)	
Positive	12 (66.7)	6 (33.3)	
HER2			1
Negative	8 (47.1)	9 (52.9)	
Positive	11 (50.0)	11 (50.0)	
Ki-67 labeling index			0.407
< 20%	4 (66.7)	2 (33.3)	
≥ 20%	15 (45.5)	18 (54.5)	
Stromal TILs			0.03
Non-LPBC	17 (58.6)	12 (63.1)	
LPBC	2 (20.0)	8 (80.0)	
FOXP3			0.32
Low	11 (42.3)	15 (57.7)	
High	8 (61.5)	5 (38.5)	
eTreg			0.082
Low	11 (39.3)	17 (60.7)	
High	8 (72.7)	3 (27.3)	
CD8			0.02
Low	18 (60.0)	12 (40.0)	
High	1 (11.1)	8 (88.9)	
CD8/FOXP3			0.007
Low	16 (66.7)	8 (33.3)	
High	3 (20.0)	12 (80.0)	
CD8/eTreg ratio			0.001
Low	18 (66.7)	9 (33.3)	
High	1 (8.3)	11 (91.7)	

*ER* estrogen receptor, *HER2* human epidermal growth factor receptor 2, *LPBC* lymphocyte predominant breast cancer, *TILs* tumor-infiltrating lymphocytes

**Table 4:** Logistic regression analysis for predicting pathological complete response

	Univariate analysis		Multivariate analysis	
	OR (95% CI)	*P*	OR (95% CI)	*P*
ER-positive	0.25 (0.07–0.95)	0.042	0.17 (0.02–1.19)	0.074
HER2-positive	0.89 (0.25–3.16)	0.855	0.32 (0.04–2.44)	0.274
Ki-67 labeling index ≥ 20%	2.40 (0.39–15.0)	0.349	1.19 (0.11–12.6)	0.884
LPBC	10.4 (1.88–57.4)	0.007	10.1 (1.08–94.2)	0.043
CD8/eTreg ratio high	22.0 (2.44–198)	0.006	18.7 (1.25–279)	0.034

*CI* confidence interval, *ER* estrogen receptor, *HER2* human epidermal growth factor receptor 2, *LPBC* lymphocyte predominant breast cancer, *OR* odds ratio

**Fig. 2:**
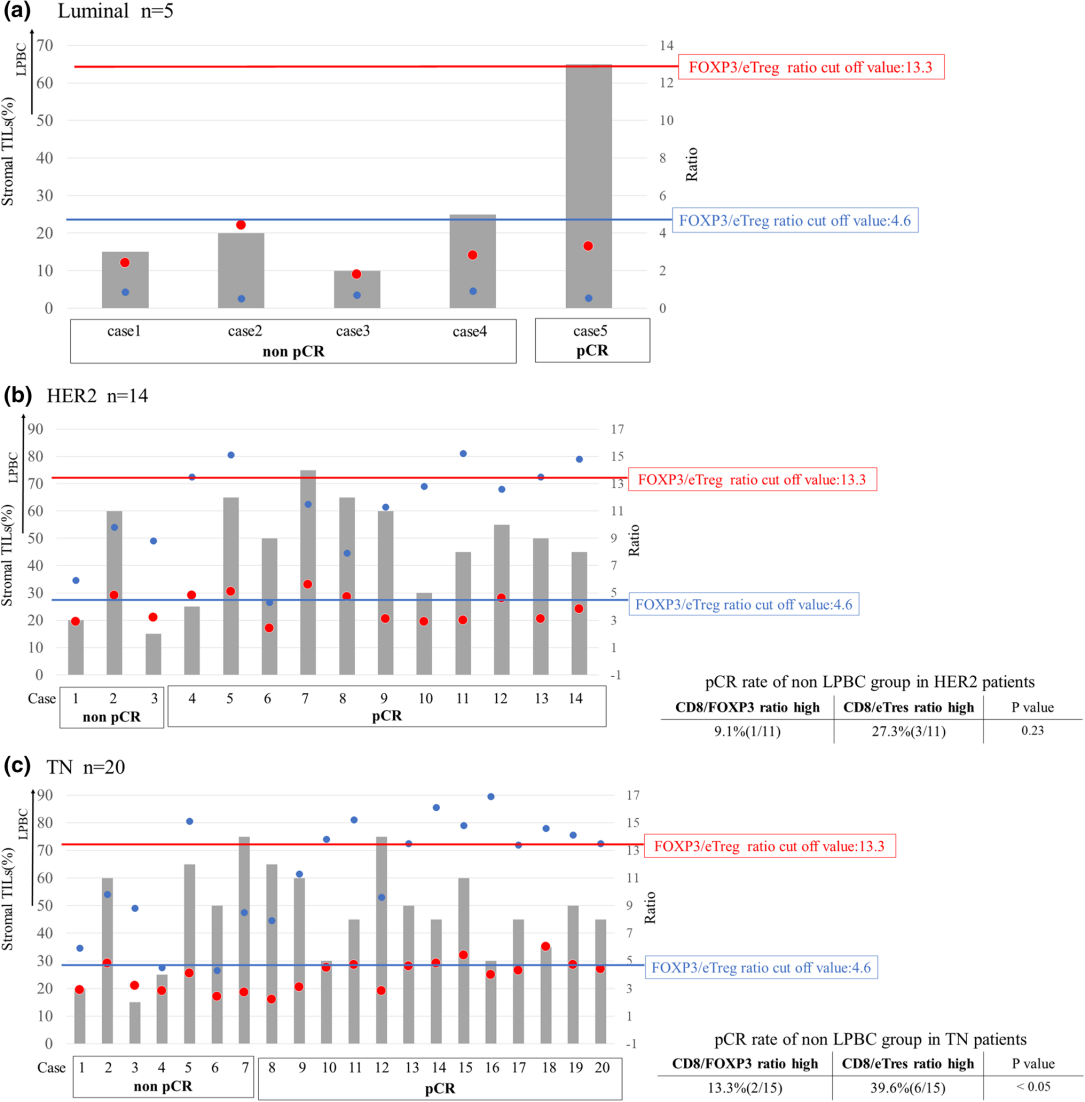
Detailed data of patients who underwent NAC of **a** luminal, **b** HER2, and **c** TN subtypes. The stroma TIL, CD8/FOXP3 + ratio (blue dot, cut off value: 4.6), CD8/eTreg ratio (red dot, cut off value;13.3), and treatment response (pCR or non pCR) are shown in each case. *pCR* pathological compete response, *TN* triple negative

We carried out a detailed assessment of the CD8/eTreg ratio for each patient who underwent NAC because pCR is empirically associated with LPBC (Fig. 2). We focused on non-LPBC patients who achieved pCR. HER2 patients with a high CD8/eTreg ratio tended to have a higher pCR ratio compared to patients with a low CD8/FOXP3 ratio (pCR rate: CD8/eTreg ratio 27.3% vs CD8/eTreg ratio 9.1%, P = 0.23). TN patients with a high CD8/eTreg ratio had a significantly higher pCR rate compared to patients with a low CD8/FOXP3 ratio (39.6% vs 13.3, P < 0.05) (Fig. 2b, c). We were not able to analyze the luminal subtype because of the number of cases was insufficient.

## Discussion

In this study, we focused on the immunological functional heterogeneity of FOXP3 + TILs and evaluated the balance of eTreg and CD8 TILs as representative of the immunoprogressive and immunosuppressive power of TILs in patients with breast cancer. We aimed to assess eTregs in breast cancer TILs and verify the association between the functional balance of TILs and clinical outcome of breast cancer. We demonstrated the functional heterogeneity of FOXP3 + Tregs using fresh TIL samples. Tregs are components of CD4 + T cells and essential effector cells that maintain immune homeostasis [9, 10, 36–38]. Although Tregs are considered to be homogeneously immunosuppressive TILs in breast cancer, in this study, we demonstrated the strong suppressive function of eTregs and the non-suppressive function of other Treg subpopulations. We found that eTreg cells were more abundant in breast cancer tissues than in PBMCs and LNBTs, which is consistent with previous reports on other cancer types, such as colorectal [31] and gastric cancer [37]. The difference in the infiltration of Treg subpopulations indicates that T cells are activated and acquire functions in the tumor microenvironment. It is worth noting that patients with low levels of eTreg to total FOXP3 + TILs can overestimate their immunosuppressive function by the FOXP3 immunohistochemical staining method. Thus, these patients may lead to conflicting results in some studies that are based on immunohistochemical evaluation of FOXP3 for breast cancer prognosis. LPBC is considered as a strong biomarker for pathological response to NAC regardless of molecular subtypes [4, 6] and survival in triple-negative and HER2 + subtype [5, 38]. Our findings indicate that the CD8 + /eTreg ratio is a more sensitive predictor of pCR compared to the CD8 + /FOXP3 + ratio, especially in TN subtypes.

It has been reported that CD15s antibodies can specifically identify eTreg cells [39]. Recently, eTregs were identified in diffuse large B cell lymphoma using dual immunostaining of FOXP3 and CTLA4 [32]. Dual immunohistochemical staining methods may be a promising method for the evaluation of eTreg infiltration with a large sample size in breast cancer.

Despite the findings of this study, it has several limitations. First, the number of cases was small, and the follow-up period was short. Our findings cannot elucidate the mechanism underlying increased eTregs in the tumor microenvironment. Moreover, we did not evaluate the other TIL components. Recently, an exhaustive and promising evaluation of breast cancer TILs by single-cell RNA sequencing has been reported [40]. The genomic background or mechanism that causes these variations in TIL components will be elucidated in the near future. In addition, eTregs express immune checkpoint molecules, such as cytotoxic T-lymphocyte-associated protein 4 (CTLA-4) and programmed cell death-1 (PD-1), suggesting that controlling tumor-infiltrating Tregs may be a potential target for cancer immunotherapy [41–44]. Our findings may be linked to other studies evaluating the response to immune checkpoint inhibitors in patients with breast cancer to build on their significance.

In summary, our study indicates that the functional heterogeneity of FOXP3 + TILs represents specific variations in the immunological balance in invasive breast cancer. A high CD8/eTreg ratio enhances the treatment response in patients with invasive breast cancer, especially in patients with non-LPBC and TN subtypes. Further studies are warranted to validate these findings.

## Conclusion

We clarified Treg subpopulations TILs and balance between them and CD8 + TILs. A high CD8/eTreg ratio enhances the treatment response in patients with invasive breast cancer, especially in patients with non-LPBC and TN subtypes.

## Data Availability

The datasets generated and/or analyzed during the current study are available from the corresponding author on reasonable request.

## Abbreviations

CFSE: Carboxyfluorescein succinimidyl ester
ER: Estrogen receptor
HER2: Human epidermal growth factor receptor type2
TN: Triple negative
LPBC: Lymphocyte predominant breast cancer
OR: Odds ratio
PBMCs: Peripheral blood mononuclear cells
TILs: Tumor-infiltrating lymphocytes
